# Editorial: A sweet deal – Glycobiology in host-pathogen interactions

**DOI:** 10.3389/fmicb.2023.1341820

**Published:** 2023-12-12

**Authors:** Kunio Kawanishi, Yuko Naito-Matsui, Livia Soares Zaramela, Nina M. van Sorge, Masaya Yamaguchi

**Affiliations:** ^1^Department of Experimental Pathology, Institute of Medicine, University of Tsukuba, Tsukuba, Ibaraki, Japan; ^2^Department of Molecular Cell Biology, School of Medical Sciences, Fujita Health University, Toyoake, Aichi, Japan; ^3^Department of Biochemistry and Immunology, Ribeirão Preto Medical School, University of São Paulo, Ribeirão Preto, Brazil; ^4^Department of Medical Microbiology and Infection Prevention, Amsterdam UMCs Location University of Amsterdam, Amsterdam, Netherlands; ^5^Netherlands Reference Laboratory for Bacterial Meningitis, Amsterdam UMCs Location AMC, Amsterdam, Netherlands; ^6^Bioinformatics Research Unit, Osaka University Graduate School of Dentistry, Osaka, Japan; ^7^Department of Microbiology, Osaka University Graduate School of Dentistry, Osaka, Japan; ^8^Bioinformatics Center, Research Institute for Microbial Diseases, Osaka University, Osaka, Japan; ^9^Center for Infectious Diseases Education and Research, Osaka University, Osaka, Japan

**Keywords:** FUT2, FUT3, phenolic glycolipids, poly-N-acetylglucosamine, infection

Glycans are essential biomolecules for all life forms. Often, glycans are attached to other biomolecules such as proteins or lipids to diversify their function. As such, glycosylation represents a crucial post-translational modification with important roles in various biological recognition events (Gagneux et al., 2022). A notable aspect of functional glycan-mediated molecular recognition manifests in the interaction between the host's immune system and microorganisms during infection and symbiosis. Glycans intimately participate in each phase of the infection process (Lewis et al., 2022). Therefore, understanding the role of glycan-mediated interactions in symbiosis and pathogenesis is essential for the development of strategies to prevent and treat infectious diseases.

Within this *Frontiers in Microbiology* Research Topic, we have curated four original research papers. Two of these studies focused on polymorphisms in host glycolytic enzymes related to the susceptibility or protection to rotavirus infection, which is the most common cause of acute gastroenteritis in children under 5 years of age globally. Godefroy et al. analyzed a cohort of young healthy adults focusing on the *FUT2* gene, encoding the fucosyltransferase FUT2. FUT2 contributes to the synthesis of glycan fucosylation, which are often used as attachment factors by pathogens such as noroviruses and rotaviruses. The authors focused on the observations that a functional allele of FUT2, generating a secretory phenotype, confers susceptibility to rotavirus infection, while protecting against inflammatory and autoimmune diseases. Using anti-rotavirus antibody (anti-RVA) titers as indication for prior rotavirus infection, the authors observed that individuals with a secretory phenotype were overrepresented among individuals that had both neutralizing antibodies and high serum IgA titers. Interestingly, individuals with a secretory phenotype had higher frequencies of microbiota-induced regulatory T cells (DP8α Tregs), which appear to protect against intestinal inflammation, and higher abundance of the clostridium species *Faecalibacterium prausnitzii* in their feces. These data suggested that prior symptomatic RVA infection, which is associated with the FUT2 phenotype, may induce the development of Tregs that later provide a balanced immunological state that protects against inflammatory diseases.

Masson et al. also focused on rotavirus infections in the context of the histo-blood group antigen (HBGA) glycans. The enzymes responsible for HBGA synthesis are highly polymorphic and each of these loci are variable across geographical areas and ethnicities. Moreover, non-functional genes of ABO, FUT2 and FUT3 have been associated with a diminished risk of rotavirus infection and effectiveness of vaccination but this association is not consistently observed in different countries or regions across the world. The authors set out to clarify this conflicting data by performing a multicenter study in the pediatric emergency department of the University Hospital of Nantes (final analysis on 200 children with RV gastroenteritis and 158 controls), Western Europe, and the Cayenne Hospital (final analysis on 49 children with RV gastroenteritis and 120 controls), French Guiana, South America. These two populations do not only have a distinct genetic background but potentially also different circulating strains. Evaluation comprised Rotavirus PCR and genotyping from stool specimens, coupled with *HBGA, FUT2, FUT3*, and ABO gene blood group comparisons. At both locations, P[8]-3.6 and P[8]-3.1 strains dominated and further analyses were focused on patients infected by P[8]-3 strains regardless of subtype. The study further confirmed that both FUT2 secretor status and FUT3 Lewis status are strongly associated with the risk of rotavirus infections caused by P[8]-3, but may not hold in case of other rotavirus strains that circulate in other countries or areas of the world.

Two additional studies in our Research Topic explore the utilization of glycans in bacteria. Gisch et al. focused on the ancestral strains of *Mycobacterium tuberculosis* complex (MTBC) Lineage 1 (L1), which are a prominent cause of tuberculosis (TB) in restricted areas of the world. Compared to MTBC strains from “modern” phylogenetic lineages, L1 strains display reduced transmission rates and decreased resistance to oxidative stress and hypoxia within the host cell micro-environment. To gain insight into the differential virulence of ancient L1 strains compared to modern MTBC lineages, the authors employed whole genome sequencing to analyze the global L1 population structure and correlate. Additionally, they correlated this to NMR-based analysis of phenolic glycolipids (PGL) synthesis, which are well-known virulence factors derived from polyketides. Phylogeographic reconstruction analysis of 312 L1 MTBC strains revealed that the lowest and highest L1 population diversity occurred among strains from Southern Africa and Eastern Africa, respectively, with Southern Asia identified as their origin region. Through novel signature SNPs, the authors redefined L1 strains sub-lineage classification, contributing to future studies. They further correlated particular L1 sublineages to distinct PGL signatures and macrophage growth profiles. In brief, the results indicate that either full PGL synthesis, or complete loss of PGL may represent an advantage for MTBC strain replication in the host. This diversity may impact *M. tuberculosis* virulence and host interactions, possibly influencing disease outcomes and immune responses in different populations. Understanding these variations could have implications for diagnostics, vaccine development, and therapeutic strategies for tuberculosis caused by Lineage 1 strains. Further research may elucidate the precise roles of PGLs in *M. tuberculosis* pathogenicity and host adaptation within this lineage.

Kutsuno et al. focused on biofilms, which represent communities of microbial cells enclosed in a matrix of extracellular substances. *Staphylococcus aureus*, a well-known biofilm-forming bacterial pathogen, synthesizes a key biofilm component, poly-N-acetylglucosamine (PNAG), through machinery encoded by the *icaADBC* operon. The authors previously identified a novel negative regulator of biofilm formation, Rob, which binds to a specific 5-bp motif in the intergenic region between the *icaADBC* operon and its repressor gene, *icaR* (Yu et al., 2017). Deletion of this motif resulted in excessive adherent biofilm formation.

In a pursuit to further characterize the physiological role of the 5-bp motif, they generated specific deletion mutants, which showed an unexpected phenotype of vortex-induced self-aggregation and subsequent sedimentation in culture. Whole genome sequencing revealed the presence of unselected mutations in *icaB*, resulting in the production of non-deacetylated PNAG. Generation of a double-deficient strain, harboring mutations in biofilm inhibitory factors (5-bp motif, *icaR, rob*) and *icaB* validated that the aggregation phenomenon was unique to the Δ5bpΔ*icaB* double mutant. This study suggests that deacetylation of PNAG by IcaB is necessary for biofilm adherence and conversely demonstrates a novel mechanism for non-adherent biofilm formation in *S. aureus*.

Overall, the four papers in our Research Topic provide an exhilarating augmentation to glycobiology, shedding light on host-pathogen interactions. We trust that these contributions will propel researchers in the field toward further advancements in microbiology and glycobiology.

## Author contributions

KK: Writing – original draft, Writing – review & editing. YN-M: Writing – original draft, Writing – review & editing. LS: Writing – original draft, Writing – review & editing. NS: Writing – original draft, Writing – review & editing. MY: Writing – original draft, Writing – review & editing.
